# Eco-Friendly Filtrate Control in Drilling Fluids: Itaconic Acid-Grafted Corn Starch from Natural Organic Materials with Thermal and Salt/Calcium Resistance

**DOI:** 10.3390/polym18020244

**Published:** 2026-01-16

**Authors:** Bin Wang, Junyi Liu, Zhongwen Song

**Affiliations:** 1 Shale Oil Project Department, Shengli Oilfield Company, SINOPEC, Dongying 257015, China; wangbin738.slyt@sinopec.com (B.W.); songzhongwen.slyt@sinopec.com (Z.S.); 2 Shengli Petroleum Engineering Corporation Limited, SINOPEC, Dongying 257015, China

**Keywords:** itaconic acid, graft copolymerization, corn starch, fluid loss reducer, drilling fluid, environmentally friendly

## Abstract

This study developed a bio-based fluid loss reducer based on itaconic acid-grafted corn starch (IACS) for water-based drilling fluid systems. The product was synthesized through free radical graft copolymerization and characterized by FTIR, TGA, and SEM. In bentonite-based mud systems, IACS demonstrated excellent filtration control performance significantly superior to that of conventional fluid loss reducers such as PAM, CMC, and PAC. IACS exhibited outstanding temperature resistance, salt tolerance, and calcium contamination resistance. Particle size analysis revealed that IACS effectively dispersed bentonite particles to the nanoscale at elevated temperatures, preventing thermal aggregation. Mechanistic studies indicated that carboxyl groups introduced by the grafting reaction endowed IACS with strong adsorption capacity and hydration ability, forming a dense polymer network layer on clay particle surfaces. Environmental evaluation confirmed that IACS possessed moderate biodegradability and extremely low toxicity, meeting green drilling fluid additive requirements. This study provides new insights for developing high-performance, environmentally friendly fluid loss reducers.

## 1. Introduction

Drilling fluid is a critical working fluid in oil and gas exploration and development, performing multiple functions including maintaining wellbore stability, carrying cuttings, cooling and lubricating drill bits, and controlling formation pressure [1,2,3]. Among various performance indicators, fluid loss control is one of the core factors determining the success or failure of drilling operations. Excessive invasion of drilling fluid filtrate into formations can cause serious engineering problems such as wellbore instability, formation damage, and pipe sticking, directly affecting drilling efficiency and hydrocarbon recovery rates [4,5]. Therefore, developing efficient fluid loss reducers is crucial for ensuring drilling safety and economic viability.

Traditional fluid loss reducers such as polyacrylamide (PAM), carboxymethyl cellulose (CMC), and polyanionic cellulose (PAC), although exhibiting excellent performance, generally suffer from the following limitations: (1) petroleum-based raw materials that do not align with sustainable development principles; (2) poor biodegradability with environmental pollution risks; (3) performance deteriorates sharply under extreme downhole conditions such as high temperature (>150 °C), high salinity (>10% NaCl), and high calcium content (>1% CaCl_2_) [6,7,8]. With increasingly stringent environmental regulations and growing demands for deep/ultra-deep drilling, developing novel fluid loss reducers that combine high performance with environmental compatibility has become an urgent need.

Natural polysaccharides have become ideal raw materials for green drilling fluid additives due to their abundant resources, biodegradability, and various modifiable functional groups [9,10,11]. Among them, starch has attracted significant attention due to its low cost, easy availability, excellent film-forming properties, and water retention capacity. However, natural starch is prone to molecular chain breakage and thermal degradation at high temperatures, limiting its application in deep well drilling [12,13]. Chemical modification, particularly graft copolymerization technology, has been proven to effectively enhance the thermal stability and functionality of starch-based materials [14,15].

Currently, monomers such as acrylic acid (AA), acrylamide (AM), and maleic anhydride (MAH) are commonly used for starch graft modification, but these monomers are mostly petroleum-based, somewhat weakening the “green” attributes of the final products [16]. Itaconic acid (IA, chemical formula C_5_H_6_O_4_, structure CH_2_=C(COOH)CH_2_COOH), as a bio-based unsaturated dicarboxylic acid that can be prepared through carbohydrate fermentation, possesses both a reactive C=C double bond and dual carboxyl groups. This unique molecular structure provides IA with high reactivity for free radical polymerization while introducing strong hydrophilicity and ion exchange capacity to the grafted product, making it an ideal green grafting monomer [17,18]. Grafting itaconic acid onto starch molecules can significantly enhance material hydrophilicity, thermal stability, and ion exchange capacity while maintaining biodegradability.

The choice of IA over conventional petroleum-based monomers (acrylic acid, acrylamide, maleic anhydride) was driven by multiple advantages. IA is produced via microbial fermentation of renewable carbohydrates using *Aspergillus terreus*, aligning with sustainability goals. The dual carboxyl functionality provides superior hydrophilicity and electrolyte tolerance compared to single-group monomers, critical for high-salinity drilling environments. Carboxyl groups exhibit better thermal stability (>200 °C) than amide groups, which undergo hydrolysis at elevated temperatures. Moreover, IA is commercially available at the industrial scale (global production > 80,000 tons/year) with competitive pricing (USD 8–12/kg), ensuring economic viability for large-scale oilfield applications.

This study aims to prepare an environmentally friendly fluid loss reducer IACS through itaconic acid graft modification, systematically evaluate its fluid loss control performance, rheological properties, and anti-contamination capabilities under different temperature and salinity conditions, elucidate its mechanism of action, and assess its environmental safety, providing a scientific basis for developing a new generation of high-performance green drilling fluid systems.

## 2. Materials and Methods

### 2.1. Experimental Materials

Corn starch (analytical grade, Sinopharm Chemical Reagent Co., Ltd., Shanghai, China), itaconic acid (IA, purity ≥ 99%, Aladdin Biochemical Technology Co., Ltd., Shanghai, China), potassium persulfate (K_2_S_2_O_8_, purity ≥ 99%, initiator, Macklin Biochemical Technology Co., Ltd., Shanghai, China), sodium hydroxide (NaOH), hydrochloric acid (HCl), sodium chloride (NaCl), calcium chloride (CaCl_2_), and anhydrous sodium carbonate (Na_2_CO_3_) were all analytical grade and purchased from Tianjin Fuchen Chemical Reagents Factory (Tianjin, China).; bentonite was sodium-based and drilling fluid grade(obtained from Renqiu City Bohai Mud Co., Ltd., Renqiu, Hebei, China); and deionized water was used to prepare all solutions.

Commercial fluid loss reducers for comparative experiments: PAM (molecular weight 5–6 million, cationicity 5–10%), CMC (degree of polymerization 800–1000, degree of substitution 0.8–0.9) and PAC (degree of polymerization 1000–1200, degree of substitution 1.0–1.2), which were purchased from Henan Xinxiang No. 7 Chemical Co., Ltd. (Xinxiang, Henan, China).

### 2.2. Preparation of IACS

**Figure 1:**
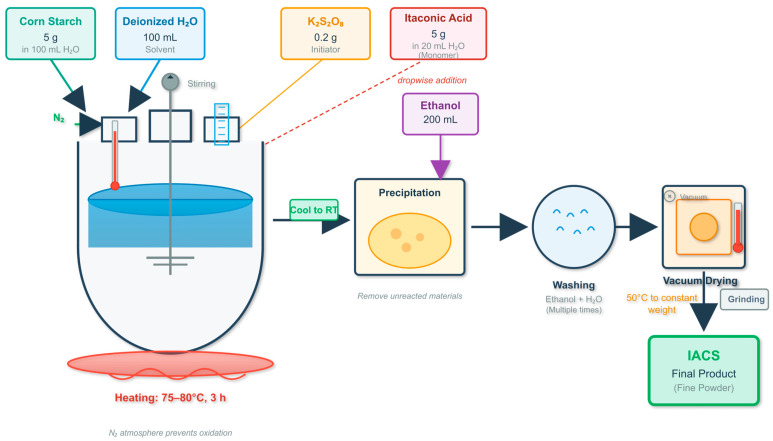
Schematic diagram of preparation process.

IACS was prepared using the aqueous solution free radical graft copolymerization method, with the process flow shown in Figure 1. Specific steps are as follows:

(1) Add 5.0 g corn starch and 100 mL deionized water to a 250 mL three-neck flask, stir to form a uniform suspension;
(2) Purge with nitrogen for 30 min to remove oxygen, heat to 75 °C and maintain temperature stability;
(3) Add 0.2 g K_2_S_2_O_8_ initiator, stir for 10 min to generate free radical active sites on starch hydroxyl groups;
(4) Dissolve 5.0 g itaconic acid in 20 mL deionized water to form a solution, add dropwise to the reaction system over 60 min;
(5) React at 75–80 °C for 3 h under continuous stirring with nitrogen protection;
(6) After reaction completion, cool to room temperature, add 200 mL anhydrous ethanol to precipitate the product;
(7) Wash the solid product alternately with ethanol and deionized water 3 times, vacuum dry (50 °C, 12 h) to constant weight;
(8) Grind into fine powder, seal and store for later use.

Control experiment: Under the same temperature and stirring conditions, prepare blank samples without initiator or monomer.

**Figure 2:**
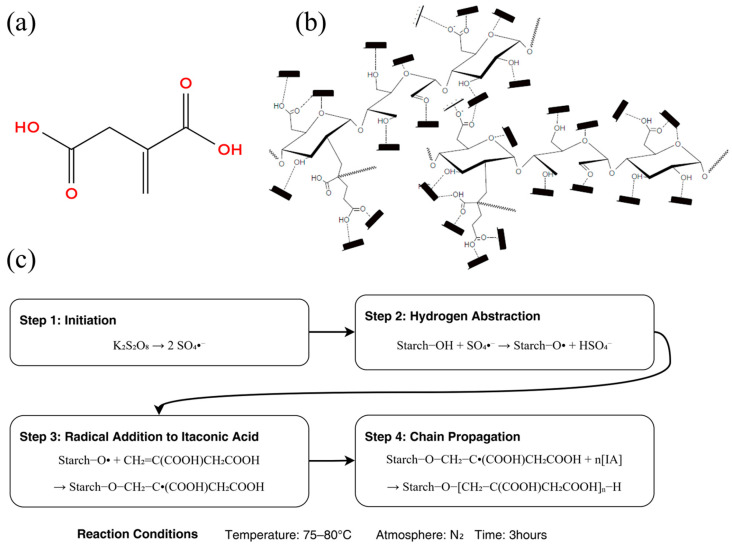
Synthesis and structure of IACS: (**a**) chemical structure of itaconic acid showing C=C double bond and dual carboxyl groups; (**b**) molecular structure of IACS showing itaconic acid grafted onto corn starch backbone; (**c**) schematic diagram of free radical graft copolymerization mechanism illustrating the four-step synthesis process (initiation, hydrogen abstraction, radical addition, and chain propagation) under the reaction conditions of K_2_S_2_O_8_ initiator, 75–80 °C, N_2_ atmosphere, and 3 h.

Theoretical reaction mechanism: Under K_2_S_2_O_8_ initiation, -OH groups on starch molecules are oxidized to free radicals (-O·), which then undergo free radical addition reactions with the C=C double bond of itaconic acid, forming graft copolymers (Figure 2). The carboxyl groups (-COOH) introduced by the grafting reaction significantly alter the physicochemical properties of starch.

### 2.3. Characterization Methods

FTIR analysis: Shimadzu IRTracer-100 Fourier transform infrared spectrometer (Shimadzu Corporation, Kyoto, Japan), test range 4000–400 cm^−1^, resolution 4 cm^−1^, 32 scans, KBr pellet method for sample preparation.

TGA analysis: TA Instruments Q50 thermogravimetric analyzer (TA Instruments, New Castle, DE, USA), sample mass 5–10 mg, nitrogen atmosphere (flow rate 50 mL/min), heating rate 10 °C/min, temperature range 25–700 °C.

SEM analysis: Hitachi SU5000 scanning electron microscope (Hitachi High-Technologies Corporation, Tokyo, Japan), acceleration voltage 5 kV, samples gold-coated (coating thickness approximately 10 nm).

### 2.4. Drilling Fluid Performance Evaluation

#### 2.4.1. Drilling Fluid Sample Preparation

Base mud formulation: 4.0% (mass fraction, hereinafter) bentonite + 0.2% Na_2_CO_3_ + deionized water to 100%. Preparation method: Disperse bentonite and Na_2_CO_3_ in 350 mL deionized water, hydrate at room temperature for 24 h to ensure complete swelling of bentonite.

IACS drilling fluid formulation: Base mud + 1.0%/2.0%/3.0% IACS. Addition method: Use high-speed shear disperser (11,000 rpm, 30 min) to uniformly disperse IACS in base mud.

Comparative drilling fluid formulation: Base mud + 2.0% PAM/CMC/PAC (consistent with optimal IACS dosage).

Salt contamination experiment formulation: Base mud + 2.0% IACS + 3%/6%/9%/12% NaCl or 0.5%/1.0%/1.5%/2.0% CaCl_2_.

#### 2.4.2. Rheological Measurement

According to API RP 13B-1 standard [19], use a six-speed rotational viscometer (Model ZNN-D6, Qingdao Haitongda Co., Qingdao, China) to measure drilling fluid rheology and record apparent viscosity at room temperature (25 °C). Evaluate thermal stability by hot-rolling samples in stainless steel aging cells using a roller oven at 180 °C for 16 h (heating rate: 10 °C/min, rotation speed: 30 rpm); then, cool to room temperature and re-measure rheological parameters.

#### 2.4.3. Filtration Performance Testing

API filtration test: Use API standard filtration apparatus to measure filtration volume over 30 min under 0.69 MPa pressure, record filtrate volume (precision ± 0.1 mL).

HTHP filtration test: Use high-temperature, high-pressure filtration apparatus, test conditions 180 °C and 3.5 MPa, measure filtration volume over 30 min. Preheat drilling fluid at test temperature for 30 min before testing.

#### 2.4.4. Temperature, Salt, and Calcium Resistance Stability

Evaluate temperature, salt, and calcium resistance stability by monitoring drilling fluid viscosity and filtration loss before and after hot rolling under different conditions. Standard thermal aging protocol: 180 °C for 16 h in roller oven unless otherwise specified. Temperature range tested: 80–200 °C; NaCl concentration: 0–12% (mass fraction); CaCl_2_ concentration: 0–2% (mass fraction).

#### 2.4.5. Particle Size Distribution Testing

Use laser particle size analyzer (Mastersizer 3000, Malvern Panalytical Ltd., Malvern, UK) to measure particle size distribution of solid phases in drilling fluids. Dilute samples to appropriate concentration before testing, ultrasonically disperse for 5 min. Measure particle size distribution of base mud and IACS/base mud after thermal aging at 25 °C and 150 °C, with 3 parallel measurements per sample group averaged.

#### 2.4.6. Environmental Performance Testing

Evaluate ecological compatibility of IACS and comparative polymers using standard environmental evaluation methods: evaluate biodegradability using BOD_5_/COD_cr_ ratio method, evaluate acute toxicity through median lethal concentration (LC_50_) testing. These tests aim to verify whether IACS can serve as a green alternative to traditional fluid loss reducers while maintaining non-toxic characteristics.

#### 2.4.7. Technological Application Performance Evaluation

To assess practical field deployment viability, IACS was compared with natural corn starch (CS) and polyacrylamide (PAM) using application-oriented performance metrics. All comparative tests used 2% additive dosage and 180 °C thermal aging (16 h) conditions.

Filter cake quality: After API filtration, filter cakes were measured for thickness (digital vernier caliper, ±0.01 mm), compactness (density = mass/volume), and permeability (mini-permeameter or calculated from re-filtration tests, mD).

Suspension capability: 10.0 g quartz sand (70–140 mesh) was added to 350 mL drilling fluid samples. After aging and cooling, sand settling rate (mm/h) was recorded in graduated cylinders over 8 h.

## 3. Results and Discussion

### 3.1. Structural Characterization

#### 3.1.1. Fourier Transform Infrared Spectroscopy (FTIR) Analysis

**Figure 3:**
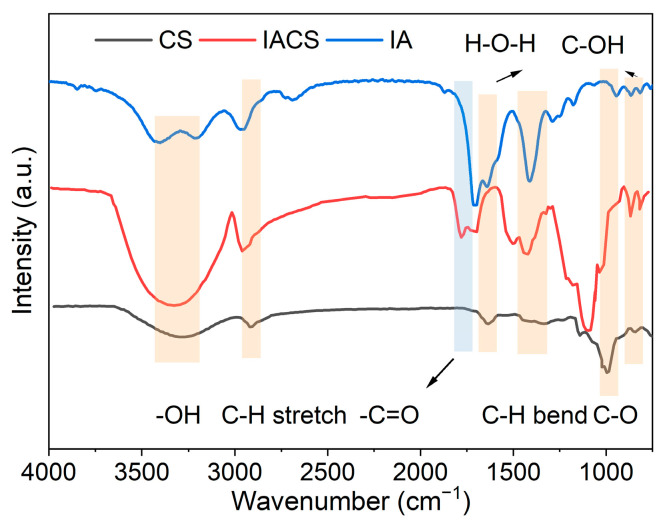
FTIR analysis.

Figure 3 shows the FTIR spectra of natural corn starch (CS), itaconic acid-grafted corn starch (IACS), and pure itaconic acid (IA). Both CS and IACS exhibit broad absorption peaks near 3400 cm^−1^, attributed to -OH stretching vibrations and hydrogen bonding; IACS shows higher absorption intensity in this region, indicating that oxygen-containing functional groups introduced by the grafting reaction enhanced the hydrogen bonding network. The C-H stretching vibration peak near 2920 cm^−1^ shows no significant difference between the two samples [20].

The IACS spectrum shows a new absorption peak at 1770 cm^−1^, attributed to ester C=O stretching vibrations (starch-O-CO-R) formed during the grafting reaction. This wavenumber is characteristic of ester groups, which appear at higher frequencies than free carboxylic acid C=O (1683 cm^−1^, expected for the conjugated C=C-COOH structure in itaconic acid) [21], directly confirming ester bond formation between starch hydroxyl groups and itaconic acid [22]. Pure itaconic acid displays its carboxylic acid C=O peak at 1710 cm^−1^ (the shift from the theoretical 1683 cm^−1^ may be due to hydrogen bonding or dimerization) and C=C stretching at 1638 cm^−1^. The appearance of the 1770 cm^−1^ ester peak in IACS, absent in both CS and pure IA, provides unequivocal evidence of chemical grafting rather than physical mixing.

Meanwhile, the absorption peak at 1630 cm^−1^ is enhanced in IACS, which may originate from H-O-H bending vibrations of bound water, C=C stretching from the vinyl group of IA, or asymmetric stretching vibrations of carboxylate (-COO^−^) formed by partial deprotonation of unreacted carboxyl groups [23].

In the fingerprint region, absorption peaks at 1440–1460 cm^−1^ correspond to C-H bending vibrations [24]; absorption peaks near 1080 cm^−1^ and 990 cm^−1^ are attributed to C-O stretching vibrations and C-OH bending vibrations, respectively [25]. Compared to CS, IACS shows increased absorption peak intensity and slight peak shape changes in the C-O region, indicating that some -OH groups participated in esterification reactions or formed new C-O-C bonds.

In summary, the appearance of a new C=O absorption peak at 1770 cm^−1^, the characteristic peaks of pure IA at 1710 and 1638 cm^−1^, and significant changes in O-H and C-O vibration regions confirm successful grafting of itaconic acid onto starch molecules, consistent with previously reported literature [26].

#### 3.1.2. Thermogravimetric Analysis

**Figure 4:**
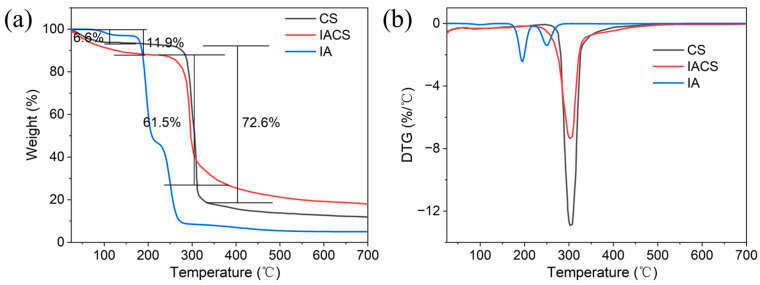
(**a**) TGA and (**b**) DTG curves of CS, IACS, and IA.

Figure 4 shows the TGA curves (Figure 4a) and DTG curves (Figure 4b) of pure itaconic acid (IA), CS and IACS. For comparison, pure IA, as a small molecular organic acid, exhibits characteristic low-temperature decomposition: it exhibits initial dehydration at 25–150 °C with ~3% weight loss, followed by rapid decomposition at 150–250 °C with a sharp DTG peak at 195 °C. By 250 °C, IA loses approximately 73% of its weight, leaving only ~5% residue at 600 °C. From the TGA curves, the thermal weight loss process of CS and IACS can be divided into three distinct stages. The first stage (25–200 °C) corresponds to the removal of physically adsorbed water and bound water, with CS showing a weight loss of 6.6% and IACS at 11.9%, the latter significantly higher. This indicates that carboxyl groups and other hydrophilic groups introduced by the grafting reaction significantly enhanced the material’s water retention capacity, enabling IACS to adsorb and bind more water molecules. The second stage (200–400 °C) is the main thermal decomposition stage, with CS showing a sharp DTG peak at approximately 300 °C, corresponding to the maximum weight loss rate, with a weight loss of 61.5% in this stage, mainly attributed to rapid breakage and dehydration reactions of starch molecular chains. In contrast, IACS shows a significantly broader and reduced DTG peak, with the peak temperature slightly shifting to higher temperatures at approximately 310 °C, and weight loss increasing to 72.6% in this stage. The third stage (400–700 °C) is the slow carbonization process of residual materials, with both samples showing low weight loss rates in this stage.

Although CS and IACS show similar decomposition temperatures (~300 °C), which is expected as both are primarily starch-based, the characteristic of IACS showing a broader and reduced DTG peak indicates that its thermal decomposition process changed from single rapid breakage to multi-step slow decomposition, which is consistent with the increased structural complexity of the graft copolymer. The elimination of IA’s characteristic 195 °C decomposition peak in IACS confirms that IA monomers are chemically grafted onto the starch backbone rather than physically mixed. This change in thermal decomposition behavior can be explained from multiple perspectives. First, grafted itaconic acid segments have different chemical structures from the starch backbone, and their decomposition pathways include various reactions such as carboxyl removal and C-C bond breakage, which occur stepwise at different temperatures, thereby dispersing the thermal decomposition energy over a wider temperature range and avoiding intense decomposition concentrated at a single temperature point [27]. Second, the grafting reaction may form slight crosslinking structures between starch molecular chains, and this crosslinking network improves the overall stability of molecular chains, making them less susceptible to rapid depolymerization at high temperatures, thereby delaying the thermal decomposition process [28]. Additionally, hydrogen bonding interactions between grafted carboxyl groups and hydroxyl groups on starch molecules can form stronger molecular internal and intermolecular forces, requiring higher energy to break these bonds.

It is worth noting that the total weight loss of IACS (72.6%) in the main thermal decomposition stage is higher than that of CS (61.5%), mainly because grafted itaconic acid segments generate more volatile small-molecule products during decomposition, such as CO_2_, CO, and organic acids, leading to increased mass loss. However, although total weight loss has increased, from a practical application perspective, the broadening of the thermal decomposition temperature range and the increase in peak temperature are of greater practical significance. For drilling fluid applications, the actual working temperature is typically below 200 °C, and IACS demonstrates better thermal stability in this temperature range, with a higher initial decomposition temperature and slower decomposition rate. Notably, at the application temperature of 180 °C, pure IA would have lost approximately 30% of its weight and would be thermally unsuitable, while IACS maintains excellent structural integrity with minimal weight loss, confirming its suitability for high-temperature drilling operations. Therefore, overall, although IACS shows a slight increase in total weight loss at high temperatures, its thermal stability within the practical application temperature range (<200 °C) is significantly superior to natural starch, better meeting the requirements of high-temperature drilling operations.

#### 3.1.3. IACS Morphological Characteristics and Effect on Base Mud Particle Size

**Figure 5:**
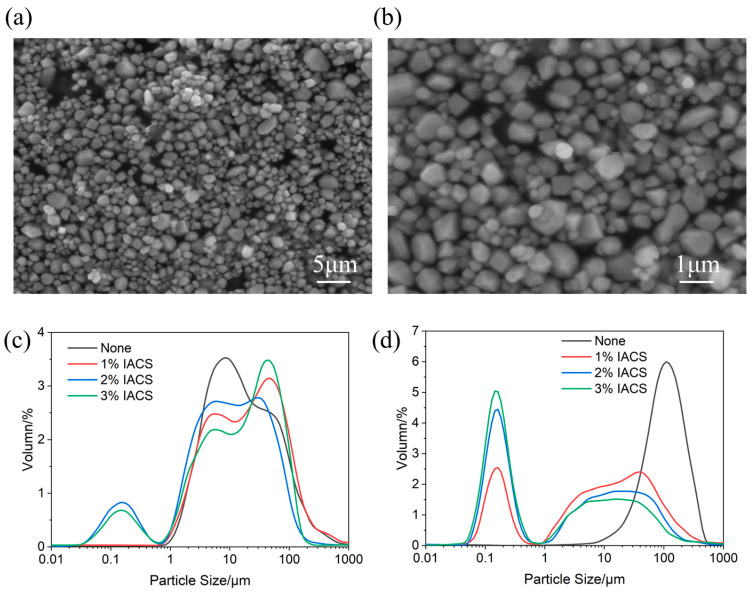
Low-magnification (**a**) and high-magnification (**b**) SEM images of IACS and particle size distribution of base mud with different IACS additions at room temperature (**c**) and 150 °C (**d**).

Figure 5a,b show SEM images of IACS. The low-magnification image (Figure 5a) shows that IACS particles are irregularly shaped, with a particle size distribution ranging from 0.5 to 2.0 μm, with an average particle size of approximately 1.0 μm. The high-magnification image (Figure 5b) shows rough particle surfaces with obvious groove and fold structures, significantly increasing the specific surface area. This micron-scale rough morphology provides IACS with abundant active sites, facilitating its adsorption and dispersion in drilling fluids; meanwhile, the rough surface enhances mechanical interlocking with bentonite particles, helping to form a dense filter cake.

Figure 5c,d show the effects of different IACS dosages on base mud particle size distribution at room temperature and 150 °C, respectively. The results indicate that IACS has a significant dispersing effect on bentonite particles, and this effect is more pronounced at high temperatures.

At room temperature, the particle size distribution main peak of blank base mud is located between 10 and 20 μm. After adding IACS, particle size distribution changes significantly: the main peak positions of 1% and 2% IACS systems remain at approximately 10 μm, but peak shapes broaden and peak values decrease, and more importantly, an obvious nanoscale particle distribution peak appears at 0.1 μm, indicating that IACS effectively dispersed some agglomerated bentonite particles to the nanoscale. The main peak of the 3% IACS system slightly increases to approximately 20 μm with the highest peak value, possibly due to slight bridging flocculation effects from excess dosage.

The particle size distribution changes at high temperature are even more significant. After thermal aging at 150 °C, the main particle size distribution peak of the blank base mud shifts significantly to approximately 100 μm with a sharp peak shape, indicating severe particle agglomeration at high temperatures, forming large aggregates. This phenomenon of dramatic particle size increase reflects that high temperatures severely weaken the hydration and dispersion capacity of bentonite, leading to reduced electrostatic repulsion between particles and enhanced flocculation, mutually confirming the macroscopic phenomena of decreased viscosity and increased filtration loss of base mud at high temperatures. In contrast, systems with added IACS show completely different particle size distribution characteristics. The 1% IACS system presents a bimodal distribution with peaks at 0.1 μm and 10–20 μm, indicating a moderate dispersion effect. The 2% IACS system shows the most outstanding dispersion effect, with the main peak significantly shifting to 0.1 μm (100 nm) with a sharp and concentrated peak shape, while only a smaller secondary peak exists at 10 μm. This particle size distribution indicates that 2% IACS can still disperse most bentonite particles to the nanoscale at high temperatures, representing a particle size reduction of 99.9% compared to the 100 μm large aggregates of blank base mud, fully demonstrating IACS’s excellent high-temperature dispersion capability. The main peak of the 3% IACS system is located at approximately 10 μm, with a dispersion effect inferior to 2% IACS, further verifying that 2% is the optimal dosage.

### 3.2. Performance Characterization

#### 3.2.1. Environmental Performance Analysis

Table 1 lists the environmental performance parameters of IACS and CS. The LC_50_ of IACS reaches 81,600 mg/L, significantly higher than the toxicity threshold (1000 mg/L), indicating extremely low acute toxicity and meeting environmental safety requirements. The BOD_5_/CODcr ratio is 0.35, falling into the moderately biodegradable category, slightly better than natural starch (0.31).

Grafted carboxyl groups increase material hydrophilicity and surface active sites, promoting microbial attachment and oxidative metabolism. Although the grafting reaction may form some difficult-to-degrade C-C covalent bonds, the overall biodegradability remains at an acceptable level, far superior to that of synthetic polymers such as PAM (BOD_5_/COD_cr_ < 0.1).

#### 3.2.2. Filtration Performance Analysis

**Figure 6:**
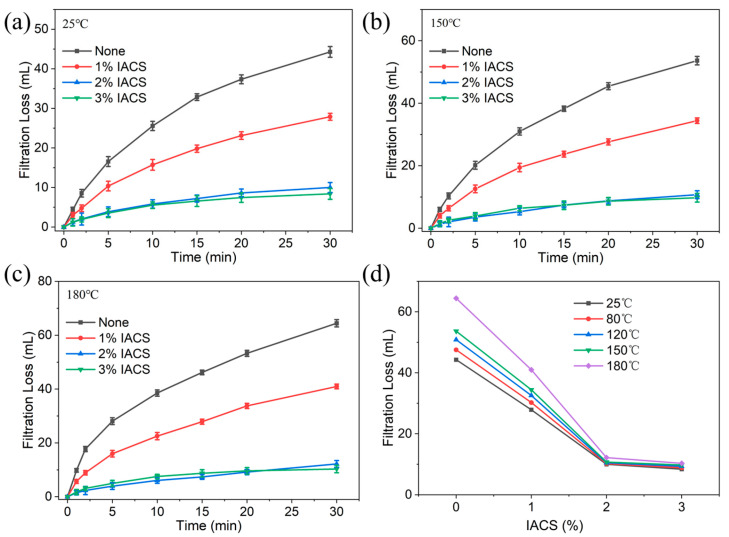
Effect of IACS concentration on the filtration loss of bentonite base slurry at different temperatures after 180 °C/16 h thermal aging. (**a**) 25 °C; (**b**) 150 °C; (**c**) 180 °C; (**d**) filtration loss after 30 min at various temperatures.

Figure 6 shows the effects of different IACS concentrations on bentonite base mud filtration loss at different temperatures. All samples were thermally aged at 180 °C for 16 h in a roller oven according to the API RP 13B-1 standard before testing. At 25 °C, room temperature (Figure 6a), the 30 min filtration loss of blank base mud is 44.2 mL, showing poor filtration control capabilities. After adding IACS, filtration loss decreases significantly with obvious concentration dependence: 1% IACS reduces filtration loss to 27.8 mL, 2% IACS further reduces to 9.9 mL, and 3% IACS reduces to 8.4 mL. This indicates that even at room temperature, IACS can effectively control drilling fluid filtration loss.

As the temperature increases, IACS’s filtration loss reduction advantages become more prominent. At 150 °C (Figure 6b), the filtration loss of blank base mud sharply increases to 53.6 mL, reflecting that high temperature severely weakens bentonite’s hydration capacity and particle dispersion stability. Systems containing IACS still maintain good filtration control capability, with the filtration loss increases for different IACS concentration systems significantly smaller than those of blank base mud. When the temperature further increases to 180 °C (Figure 6c), blank base mud filtration loss increases to 64.4 mL, while filtration losses for the 1%, 2%, and 3% IACS systems are 41.0 mL, 12.2 mL, and 10.3 mL, respectively, representing reductions of 36%, 81%, and 84% compared to blank base mud. Figure 6d summarizes the relationship between filtration loss and IACS concentration across the full temperature range (25 °C, 80 °C, 120 °C, 150 °C, and 180 °C), clearly showing the trend of filtration loss decreasing with increasing IACS dosage at all tested temperatures. At intermediate temperatures (80 °C and 120 °C), the 2% IACS system maintains filtration losses of 10.2 mL and 10.5 mL, respectively, demonstrating exceptional thermal stability with minimal performance variation. Notably, the 2% IACS system shows filtration loss increasing by only 2.2 mL (from 10.0 mL to 12.2 mL) over the entire 25–180 °C range, representing an increase of merely 22%, while blank base mud shows a 46% increase (from 44.3 mL to 64.4 mL) over the same temperature range. It is worth noting that 2% IACS shows the best cost-effectiveness, still controlling filtration loss at low levels at a high temperature of 180 °C, while the filtration loss reduction from 3% IACS compared to 2% is no longer significant (less than 16%), indicating that filtration loss control efficiency has approached saturation, with diminishing economic benefits from excessive addition.

**Figure 7:**
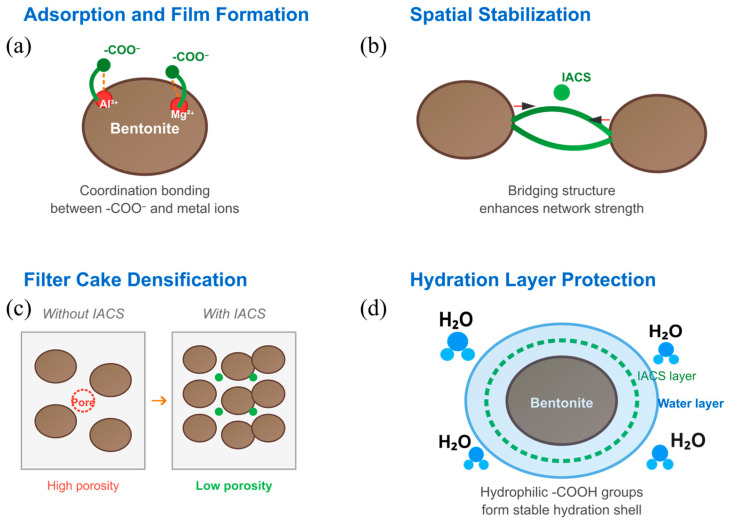
Schematic diagram of fluid loss reduction mechanisms: (**a**) adsorption and film formation; (**b**) spatial stabilization; (**c**) filter cake densification; (**d**) hydration layer protection.

IACS’s excellent filtration loss reduction performance stems from synergistic effects of multiple mechanisms. First, as shown in Figure 7a, grafted carboxyl groups can form coordination bonds with metal cations (such as Al^3+^ and Mg^2+^) on bentonite surfaces, causing IACS to firmly adsorb on particle surfaces, forming a dense polymer adsorption layer [29]. Second, as shown in Figure 7b, IACS molecular chains form bridging structures between different particles, enhancing system spatial network strength, enabling particles to arrange in an orderly manner and pack tightly during the filtration process [30]. Third, as shown in Figure 7c, IACS adsorbed on particle surfaces effectively fills pores in the filter cake, significantly reducing filter cake porosity and permeability, forming a dense physical barrier. Additionally, as shown in Figure 7d, the strong hydrophilicity of carboxyl groups promotes the formation of stable hydration layers on particle surfaces, and this hydration film further prevents rapid filtrate penetration into formations [31]. The synergistic effects of these mechanisms ensure that IACS maintains excellent filtration control capability even under high-temperature conditions.

**Figure 8:**
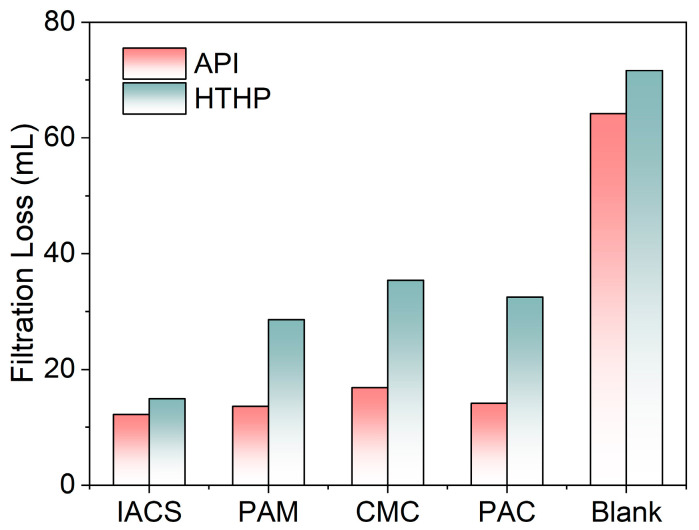
Comparison of API and HTHP filtration losses for drilling fluids containing different filtration loss control agents. Note: The base mud consists of 4 wt% bentonite and 0.2 wt% Na_2_CO_3_; aging conditions: 180 °C for 16 h in roller oven.

To further evaluate IACS’s performance advantages, Figure 8 compares the filtration performance of IACS with conventional commercial fluid loss reducers (PAM, CMC, PAC) under the same conditions. All additives were used at 2% dosage and underwent thermal aging treatment at 180 °C for 16 h before testing. The results show that in API filtration testing, IACS filtration loss is 12.2 mL, significantly lower than PAM (13.6 mL), PAC (14.1 mL), CMC (16.8 mL), and blank base mud (64.4 mL). In the more stringent HTHP filtration test, IACS’s advantages are even more significant, with an HTHP filtration loss of only 14.9 mL, while PAM, PAC, and CMC HTHP filtration losses are 28.6 mL, 32.5 mL, and 35.4 mL, respectively, with blank base mud as high as 71.6 mL. This indicates that IACS’s filtration control capability under high-temperature and high-pressure conditions far exceeds that of traditional fluid loss reducers. Moreover, IACS is synthesized from corn starch and itaconic acid, both of which are inexpensive and widely available bio-based feedstocks, offering a cost-competitive alternative to petroleum-derived fluid loss reducers.

The reasons for IACS’s performance advantages can be explained through comparison with different types of fluid loss reducers. Compared to PAM, although PAM can provide good fluid loss reduction effects at room temperature through its amide groups and long-chain structure, its molecular chains are prone to amide group hydrolysis and C-C bond breakage at high temperatures, leading to reduced molecular weight and significantly decreased fluid loss reduction capability, as can be seen from its HTHP filtration loss being significantly higher than API filtration loss. In contrast, carboxyl groups in IACS are more stable at high temperatures, less susceptible to hydrolysis reactions, and the graft copolymer structure provides additional thermal protection for the starch backbone, enabling it to maintain molecular integrity at high temperatures. Compared to CMC and PAC, these cellulose derivatives are prone to glycosidic bond breakage and decarboxylation reactions at high temperatures, leading to molecular chain breakage and functional group loss. IACS firmly fixes carboxyl groups on the starch backbone through covalent grafting, and the formed graft structure is more stable than simple etherification or esterification derivatives, ensuring its molecular integrity and functional group effectiveness under high-temperature conditions. Overall, IACS performs best in both API and HTHP filtration tests, proving its excellent filtration control capability under both ambient and high-temperature conditions, making it an ideal sustainable alternative to traditional petroleum-based fluid loss reducers.

#### 3.2.3. Rheological Analysis

**Figure 9:**
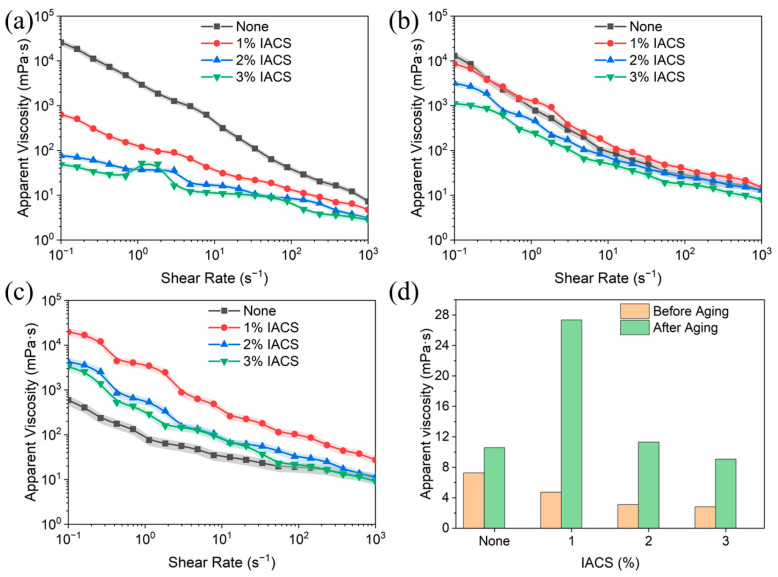
Apparent viscosity vs. shear rate with different IACS amounts added before and after 180 °C/16 h thermal aging: (**a**) 25 °C; (**b**) 150 °C; (**c**) 180 °C; (**d**) effect of IACS dosage on apparent viscosity of bentonite base mud system.

Figure 9 shows the effects of IACS dosage on bentonite base mud rheology. All systems exhibit typical pseudoplastic rheological behavior, i.e., apparent viscosity decreases with increasing shear rate, which allows drilling fluids to maintain high viscosity for suspending cuttings at low shear rates and reduce flow resistance at high shear rates.

At 25 °C, room temperature (Figure 9a), the blank base mud viscosity is approximately 20,000 mPa·s at low shear rate (0.1 s^−1^) and decreases to approximately 10 mPa·s at high shear rate (1000 s^−1^). After adding IACS, low-shear viscosity significantly decreases: 1% IACS is approximately 600 mPa·s, while 2% and 3% IACS are approximately 60 mPa·s, indicating that IACS improves bentonite dispersion. As temperature rises to 150 °C (Figure 9b), rheological curves of IACS-containing systems become more consistent with more concentrated viscosity distribution. At the high temperature of 180 °C (Figure 9c), the 1% IACS system still maintains a high viscosity of approximately 20,000 mPa·s at a low shear rate, significantly higher than other systems, demonstrating excellent high-temperature structural stability. It should be noted that the reduction in low-shear viscosity observed in IACS-containing systems at room temperature does not compromise the practical suspension capability of the drilling fluid. This is because (1) under actual drilling conditions, the drilling fluid is continuously circulated and rarely remains completely static; (2) the pseudoplastic behavior (shear-thinning) is actually desirable as it reduces pumping energy consumption during circulation while the fluid rapidly recovers structural viscosity when circulation stops; (3) most importantly, after thermal aging at high temperatures (180 °C), the IACS systems show significantly increased low-shear viscosity, indicating enhanced structural stability under downhole conditions where suspension capability is most critical.

Figure 9d compares apparent viscosity changes at a 600 rpm shear rate before and after thermal aging (180 °C × 16 h). The blank base mud viscosity increases from 7 mPa·s to 10 mPa·s after thermal aging, possibly due to irreversible particle agglomeration at high temperatures. IACS-containing systems show completely different change patterns: 1% IACS increases dramatically from 4 mPa·s to 27 mPa·s (increase over 500%), 2% IACS increases from 3 mPa·s to 11 mPa·s (increase approximately 267%), and 3% IACS increases from 2.5 mPa·s to 9 mPa·s (increase approximately 260%). The 1% IACS system shows the greatest viscosity increase, demonstrating optimal high-temperature rheological control capability. It should be emphasized that this viscosity increase after thermal aging is a highly desirable characteristic. The viscosity enhancement indicates IACS maintains molecular integrity at elevated temperatures, ensuring adequate cuttings suspension under downhole conditions while maintaining moderate viscosity at surface temperatures for efficient circulation. In contrast, conventional polymers typically show viscosity decreases after aging due to thermal degradation. This unique thermal behavior confirms the superior temperature resistance achieved through itaconic acid grafting.

IACS’s rheological improvement stems from the strong adsorption of its carboxyl groups with bentonite particles, maintaining particle dispersion and network structure even at high temperatures. Bridging structures formed by IACS molecular chains between particles are not only not destroyed at high temperatures but are strengthened through molecular chain conformation adjustment and hydrogen bond reorganization, explaining the phenomenon of viscosity increase rather than decrease after thermal aging. Combined with the particle size analysis results, the nanoscale dispersion state maintained by IACS provides a microscopic foundation for high-temperature rheological stability.

Combining filtration control and rheological regulation performance, 2% IACS is the optimal dosage formulation. It is worth noting that IACS systems show a moderate apparent viscosity at room temperature, avoiding the “low temperature–high viscosity” problem of traditional fluid loss reducers, while viscosity significantly increases at high temperatures, providing excellent structural stability. This unique rheological characteristic of “moderate at low temperature, enhanced at high temperature” enables IACS to significantly improve the high-temperature rheological stability without increasing circulation pressure losses, which has important practical value for deep well and ultra-deep well drilling operations.

#### 3.2.4. Salt and Calcium Resistance Performance Analysis

**Figure 10:**
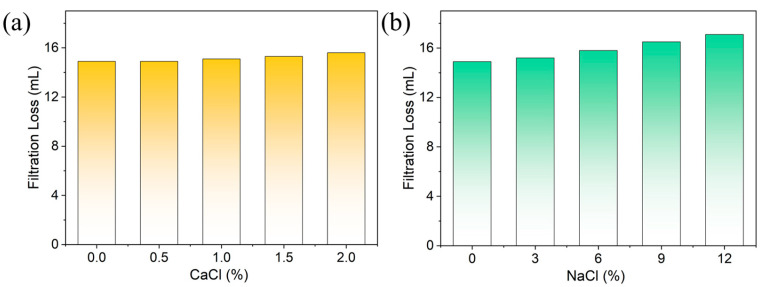
Filtration performance of IACS-based drilling fluid under different salt contamination conditions after 180 °C/16 h thermal aging: (**a**) effect of CaCl_2_ concentration; (**b**) effect of NaCl concentration.

Figure 10 shows the HTHP filtration performance of IACS/base mud (2% IACS) under different salt contamination conditions (thermal aging at 180 °C for 16 h). Salt and calcium resistance capability is a key indicator for evaluating the application potential of fluid loss reducers in high-mineralization formations.

Calcium resistance: As the CaCl_2_ concentration increases from 0 to 2%, the HTHP filtration loss remains within 14.9–15.6 mL (increase of only 5%), demonstrating excellent resistance to Ca^2+^ contamination (Figure 10a). This is particularly significant because Ca^2+^, as a divalent cation, typically causes severe performance degradation in anionic polymers through crosslinking and precipitation. Traditional fluid loss reducers such as PAM and CMC often show filtration loss increases exceeding 100% under similar Ca^2+^ concentrations.

Salt resistance: As NaCl concentration increases from 0 to 12%, HTHP filtration loss gradually increases from 14.9 mL to 17.1 mL (increase of 15%), maintaining relatively stable filtration control (Figure 10b). Conventional anionic polymers typically fail when the NaCl concentration exceeds 6%, showing filtration loss increases over 50%. IACS’s ability to maintain performance under extreme salinity (12% NaCl ≈ 200 g/L) demonstrates excellent salt tolerance.

**Figure 11:**
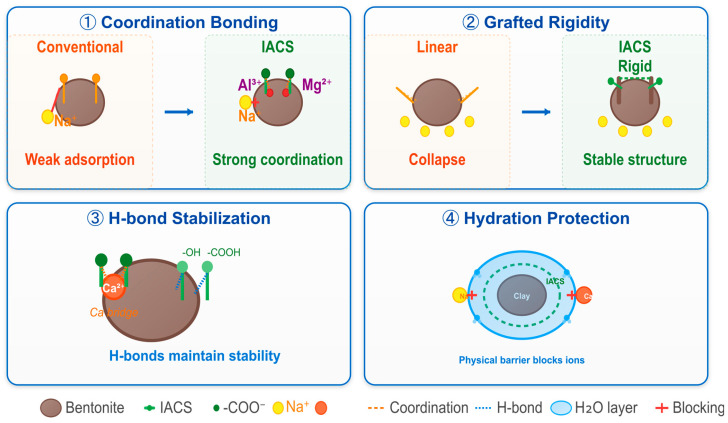
Schematic diagram of salt resistance mechanism.

IACS’s excellent salt and calcium resistance performance stems from its unique molecular structural characteristics, as shown in Figure 11. First, IACS molecules contain both ionizable carboxyl groups (-COO^−^) and numerous non-ionized carboxyl and hydroxyl groups (-COOH, -OH), and this amphoteric characteristic enables it to adsorb on bentonite surfaces simultaneously through electrostatic and coordination interactions. Coordination bonds formed between carboxyl groups and metal cations (Al^3+^, Mg^2+^) on bentonite surfaces have strong binding energy and are not easily displaced by Na^+^ or Ca^2+^. Second, IACS’s graft copolymer structure imparts a certain rigidity to molecular chains, reducing conformation collapse under high ionic strength. When the Na^+^ concentration increases, although it compresses IACS’s electrical double layer thickness and weakens electrostatic repulsion, coordination bonds and hydrogen bonding interactions can still maintain the stable adsorption of IACS on particle surfaces [32]. Third, although Ca^2+^ forms “calcium bridges” with some -COO^−^, numerous -OH and non-ionized -COOH in IACS can provide additional adsorption stability through hydrogen bonding, and the graft structure effectively prevents excessive crosslinking and precipitation induced by Ca^2+^. Additionally, the strong hydrophilicity of carboxyl groups promotes the formation of stable hydration layers on particle surfaces, physically isolating the direct contact of salt ions with particle surfaces, further maintaining system dispersion stability.

A comprehensive comparison of Figure 10a,b reveals that IACS’s resistance to Ca^2+^ is even better than its resistance to Na^+^, which is completely opposite to the behavior of traditional anionic polymers. This unique performance is attributed to IACS’s multi-point adsorption mechanism and synergistic protective effects of the graft structure. Therefore, even under extreme conditions of high temperature, high salt, and high calcium, IACS can maintain good filtration control capability, laying a solid foundation for its application in high-mineralization formations such as offshore drilling and salt dome drilling.

#### 3.2.5. Technological Application Performance Comparison

To evaluate field deployment viability, IACS was systematically compared with natural corn starch (CS) and industrial-standard polyacrylamide (PAM) using practical application metrics. Table 2 summarizes key technological performance indicators after 180 °C thermal aging.

IACS demonstrated a superior filter cake quality with 56% thinner cakes and 84% lower permeability compared to CS, directly translating to reduced formation damage and differential sticking risks in field operations. The highly compact filter cake structure (1.42 g/cm^3^) indicates efficient particle packing and polymer network formation, creating an effective barrier against fluid invasion. In contrast, thermally degraded CS produced loose, highly permeable cakes unsuitable for high-temperature applications.

Suspension capability testing showed that IACS maintained an excellent cuttings transport capacity (3.2 mm/h settling rate) after high-temperature aging, comparable to PAM (3.8 mm/h) and significantly superior to CS (18.5 mm/h). This performance ensures adequate hole cleaning in vertical and deviated wells, preventing cuttings accumulation that can lead to pack-off and stuck pipe incidents. The superior suspension capability stems from IACS’s thermal stability and ability to maintain nanoscale particle dispersion even at elevated temperatures, as confirmed by the particle size analysis (Section 3.1.3).

The economic analysis indicates that the cost of IACS (CNY 145/m^3^ mud) is competitive with that of PAM (CNY 168/m^3^) despite the additional grafting synthesis step. When considering waste disposal costs, IACS offers 25–35% total lifecycle cost savings due to biodegradability (BOD_5_/COD = 0.35), eliminating expensive hazardous waste treatment required for synthetic polymers. The use of readily available corn starch and bio-based itaconic acid as raw materials further enhances economic viability and supply chain sustainability.

These comprehensive comparative results demonstrate that IACS achieves an optimal balance of technical performance, operational stability, and economic viability. A comparison with recently reported bio-based fluid loss reducers reveals IACS’s competitive advantages: Kong et al. [12] developed a modified starch-polyamine system capable of 180 °C operation with HTHP filtration loss of 11–15 mL and salt tolerance up to 10% NaCl, but this required a complex multi-component formulation including modified starch, polyamine inhibitor, and multiple additional additives working synergistically. Long et al. [33] reported that carboxymethylated plant press slag (CMCS) achieved an excellent low-temperature filtration performance (5.3 mL at 120 °C) with good salt resistance, but its limited thermal stability restricted its application to 120 °C with poor calcium tolerance. In comparison, IACS achieves comparable or superior performance (API FL 12.2 mL, HTHP FL 14.9 mL at 180 °C, 12% NaCl tolerance, 2% CaCl_2_ tolerance) through simple one-step aqueous synthesis without requiring co-additives, representing a significant advantage in both performance and operational simplicity for industrial-scale production.

## 4. Conclusions

This study successfully developed a bio-based fluid loss reducer (IACS) through itaconic acid grafting modification of corn starch. Structural characterization confirmed the successful incorporation of carboxyl functional groups, enhanced thermal stability, and favorable morphological characteristics.

IACS demonstrated superior filtration control performance compared to conventional fluid loss reducers (PAM, CMC, PAC), maintaining effectiveness under extreme conditions including high temperature, high salinity, and calcium contamination. Mechanistic studies revealed that IACS achieves nanoscale dispersion of bentonite particles at elevated temperatures, preventing thermal aggregation and maintaining system stability through strong adsorption and hydration capabilities.

A distinctive feature of IACS is its unique “moderate at low temperature, enhanced at high temperature” rheological behavior, which is particularly advantageous for deep well drilling operations by reducing surface circulation costs while ensuring adequate downhole suspension capability. Comparative technological evaluation confirmed IACS’s practical advantages in formation protection, hole cleaning, and operational stability.

Industrial production feasibility: The synthesis employs straightforward aqueous free radical graft copolymerization under moderate conditions (75–80 °C, atmospheric pressure) using commercially available corn starch and bio-based itaconic acid. The process requires only conventional equipment available in existing starch modification facilities, with standard purification procedures (ethanol precipitation, water washing, vacuum drying). This combination of simple chemistry, established supply chains, and compatibility with existing infrastructure ensures cost-effective commercial-scale manufacturing without significant capital investment.

Environmental and economic viability: IACS exhibits moderate biodegradability and extremely low toxicity, meeting green drilling fluid requirements. Economic analysis demonstrates competitive lifecycle costs compared to petroleum-based alternatives, with 25–35% savings when including waste disposal costs. IACS presents a viable sustainable alternative to conventional fluid loss reducers, with broad application prospects in high-temperature and high-salinity drilling environments.

## Figures and Tables

**Table 1:** Evaluation of degradability of ICAS and CS.

Sample	BOD_5_ (mg/L)	COD_cr_ (mg/L)	BOD_5_/COD_cr_	LC_50_ (mg/L)
IACS	9480	27,100	0.35	81,600
CS	8770	28,400	0.31	80,400

**Table 2:** Comparative technological application performance after 180 °C/16 h thermal aging.

Performance Indicator	IACS	CS	PAM	Field Requirement
Filter cake thickness (mm)	2.1 ± 0.2	4.8 ± 0.3	2.9 ± 0.2	<3.0
Cake compactness (g/cm^3^)	1.42 ± 0.08	0.95 ± 0.12	1.28 ± 0.09	>1.2
Cake permeability (mD)	0.31 ± 0.04	1.89 ± 0.15	0.58 ± 0.07	<0.5
Sand settling rate (mm/h)	3.2 ± 0.3	18.5 ± 1.8	3.8 ± 0.4	<5.0
Relative cost (CNY/m^3^ mud)	145	95	168	Economic

## Data Availability

The data is contained within the article.
